# Epidemiology of Untreated Psychoses in 3 Diverse Settings in the Global South: The International Research Program on Psychotic Disorders in Diverse Settings (INTREPID II)

**DOI:** 10.1001/jamapsychiatry.2022.3781

**Published:** 2022-11-16

**Authors:** Craig Morgan, Alex Cohen, Georgina Miguel Esponda, Tessa Roberts, Sujit John, Joni Lee Pow, Casswina Donald, Bola Olley, Olatunde Ayinde, Joseph Lam, Paramasivam Poornachandrika, Paola Dazzan, Fiona Gaughran, Palaniyandi Ponnusamy Kannan, Selvaraju Sudhakar, Jonathan Burns, Bonginkosi Chiliza, Ezra Susser, Helen A. Weiss, Robin M. Murray, Thara Rangaswamy, Oye Gureje, Gerard Hutchinson, and the INTREPID Group, Adejoke Agboola, Olawoye Fadahunsi, Olufemi Idowu, Clement Obuene, Akin Ojagbemi, Bamise Olayiwola, Seyi Owoeye, Kulandaiyesu Amaldoss, Jothi Ramadoss Aynkaran, Abirami Balashanmugam, Premalatha Chockalingam, Kruthika Devanathan, Subhashini Gopal, Ramesh Kumar, Padmavati Ramachandran, Karthick Samikannu, Darielle Bharath-Khan, Donella Jadoo, Elysse Marcellin, Elena Raymond, Grace Sooknanan, Lauren Subnaik, Diana Williams

**Affiliations:** 1 Health Service and Population Research Department, Institute of Psychiatry, Psychology & Neuroscience, King’s College London, London, United Kingdom; 2 ESRC Centre for Society and Mental Health, Institute of Psychiatry, Psychology and Neuroscience, King’s College London, King’s College London, London, United Kingdom; 3 National Institute for Health Research Mental Health Biomedical Research Centre at South London and Maudsley NHS Foundation Trust and King's College London, London, United Kingdom; 4 Faculty of Epidemiology and Population Health, London School of Hygiene and Tropical Medicine, London, United Kingdom; 5 Schizophrenia Research Foundation, Chennai, India; 6 Department of Psychiatry, University of the West Indies, Saint Augustine, Trinidad; 7 Department of Psychiatry, University of Ibadan, Ibadan, Nigeria; 8 Institute of Mental Health, Madras Medical College, Chennai, India; 9 Psychological Medicine, Institute of Psychiatry, Psychology & Neuroscience, King's College London, London, United Kingdom; 10 Psychosis Studies Department, Institute of Psychiatry, Psychology & Neuroscience, King’s College London, London, United Kingdom; 11 Department of Psychiatry, Rajiv Gandhi General Hospital and Madras Medical College, Chennai, India; 12 Chengelpet Medical College, Chengelpet, Tamil Nadu, India; 13 Mental Health Research Group, College of Medicine and Health, University of Exeter, Exeter, United Kingdom; 14 Department of Psychiatry, University of KwaZulu Natal, Durban, South Africa; 15 Columbia Mailman School of Public Health, Columbia University, New York, New York; 16 New York Psychiatric Institute, New York

## Abstract

**Importance:**

Less than 10% of research on psychotic disorders has been conducted in settings in the Global South, which refers broadly to the regions of Latin America, Asia, Africa, and Oceania. There is a lack of basic epidemiological data on the distribution of and risks for psychoses that can inform the development of services in many parts of the world.

**Objective:**

To compare demographic and clinical profiles of cohorts of cases and rates of untreated psychoses (proxy for incidence) across and within 3 economically and socially diverse settings in the Global South. Two hypotheses were tested: (1) demographic and clinical profiles of cases with an untreated psychotic disorder vary across setting and (2) rates of untreated psychotic disorders vary across and within setting by clinical and demographic group.

**Design, Setting, and Participants:**

The International Research Program on Psychotic Disorders in Diverse Settings (INTREPID II) comprises incidence, case-control, and cohort studies of untreated psychoses in catchment areas in 3 countries in the Global South: Kancheepuram District, India; Ibadan, Nigeria; and northern Trinidad. Participants were individuals with an untreated psychotic disorder. This incidence study was conducted from May 1, 2018, to July 31, 2020. In each setting, comprehensive systems were implemented to identify and assess all individuals with an untreated psychosis during a 2-year period. Data were analyzed from January 1 to May 1, 2022.

**Main Outcomes and Measures:**

The presence of an untreated psychotic disorder, assessed using the Schedules for Clinical Assessment in Neuropsychiatry, which incorporate the Present State Examination.

**Results:**

Identified were a total of 1038 cases, including 64 through leakage studies (Kancheepuram: 268; median [IQR] age, 42 [33-50] years; 154 women [57.5%]; 114 men [42.5%]; Ibadan: 196; median [IQR] age, 34 [26-41] years; 93 women [47.4%]; 103 men [52.6%]; Trinidad: 574; median [IQR] age, 30 [23-40] years; 235 women [40.9%]; 339 men [59.1%]). Marked variations were found across and within settings in the sex, age, and clinical profiles of cases (eg, lower percentage of men, older age at onset, longer duration of psychosis, and lower percentage of affective psychosis in Kancheepuram compared with Ibadan and Trinidad) and in rates of untreated psychosis. Age- and sex-standardized rates of untreated psychoses were approximately 3 times higher in Trinidad (59.1/100 000 person-years; 95% CI, 54.2-64.0) compared with Kancheepuram (20.7/100 000 person-years; 95% CI, 18.2-23.2) and Ibadan (14.4/100 000 person-years; 95% CI, 12.3-16.5). In Trinidad, rates were approximately 2 times higher in the African Trinidadian population (85.4/100 000 person-years; 95% CI, 76.0-94.9) compared with the Indian Trinidadian (43.9/100 000 person-years; 95% CI, 35.7-52.2) and mixed populations (50.7/100 000 person-years; 95% CI, 42.0-59.5).

**Conclusions and Relevance:**

This analysis adds to research that suggests that core aspects of psychosis vary by historic, economic, and social context, with far-reaching implications for understanding and treatment of psychoses globally.

## Key Points

**Question:**

Do the clinical and demographic profiles of individuals with (and rates of) untreated psychoses (a proxy for incidence) vary across diverse settings in the Global South?

**Findings:**

In this population-based cohort study of 1038 individuals with untreated psychoses, results suggest that there were variations across settings in the Global South in clinical, sex, and age profiles and in rates of untreated psychotic disorders (eg, high rates in Trinidad vs India and Nigeria; in African vs Indian Trinidadian).

**Meaning:**

Findings of this study add to research that suggests that core aspects of psychosis vary by economic and social context, with implications for understanding and treatment of psychoses globally.

## Teaser

This cohort study investigates if the clinical and demographic profiles of individuals with, and rates of, untreated psychoses vary across diverse settings in the Global South.

## Introduction

There are striking global inequities in our knowledge and treatment of psychotic disorders. More than 80% of the world’s population lives in the Global South, which refers broadly to the regions of Latin America, Asia, Africa, and Oceania, but less than 10% of research on psychotic disorders has been conducted in these settings.^1,2^ This evidence gap is important because the epidemiology, etiology, and outcomes of psychoses vary by context and social group.^1,3,4^ In most settings in the Global South, we lack core epidemiological evidence that can inform policy and development of accessible, humane, and effective services.

A systematic review identified 15 population-based studies of the incidence of psychoses in 9 low-and middle-income countries.^5^ Drawing on our earlier review of studies and a systematic review of all incidence studies between 2002 and 2017,^1^ we identified a further 9 studies outside of Europe, North America, and Australasia. Only 7 of these 26 studies were conducted in the past 20 years (2 in Brazil, 2 in Taiwan, 1 in Suriname, 1 in South Africa, 1 in Israel).^2^ The methods used were heterogenous, and none were conducted in more than 1 country. The World Health Organization (WHO) Determinants of Outcome of Severe Mental Disorders (DOSMeD) study, the last study that included multiple sites in the Global South, was conducted more than 40 years ago.^6^

To address these evidence gaps, we established INTREPID II (International Research Program on Psychotic Disorders in Diverse Settings), a program of research on psychoses in 3 countries (India, Nigeria, Trinidad) in the Global South.

### Aim and Hypotheses

The aim of INTREPID II is to investigate the incidence, presentation, outcomes, physical health, and impacts of untreated psychotic disorders in 3 diverse settings: Kancheepuram District, Tamil Nadu, India; Ibadan, Oyo State, Nigeria; and northern Trinidad. In this article, we describe and compare core demographic and clinical characteristics of cohorts of individuals with an untreated psychosis identified in INTREPID II and present findings on rates of untreated psychoses (to approximate incidence) across and within settings. We test 2 hypotheses: (1) demographic and clinical profiles of cases with an untreated psychotic disorder vary across settings and (2) rates of untreated psychotic disorders vary across and within settings by clinical and demographic group.

## Methods

INTREPID II, started October 1, 2017, and currently ongoing, comprises incidence, case-control, cohort, and qualitative studies of psychoses in 3 settings, based on the identification, assessment, and follow-up of cohorts of individuals with an untreated psychotic disorder (cases), of population-based matched controls, and of relatives of cases. The program was implemented in economically and socially diverse settings in 3 countries (eTable 1 in the Supplement). The catchment areas comprise urban and rural areas with populations of approximately 500 000 adults aged 18 to 64 years (eAppendix 1 in the Supplement). This study followed the Strengthening the Reporting of Observational Studies in Epidemiology (STROBE) reporting guidelines.

### Case Ascertainment

To estimate rates of untreated psychoses, we sought to identify all individuals aged 18 to 64 years with an untreated *International Statistical Classification of Diseases and Related Health Problems, Tenth Revision (ICD-10)* psychotic disorder (ie, not treated with antipsychotic medication for more than 1 continuous month) over a 2-year period in each catchment area. Individuals were excluded if there was evidence of *ICD-10*–defined moderate/severe learning disability or organic cerebral disorder. These criteria mirror those in population-based studies in the Global North.^7,8^

We used a multipronged approach to identify cases.^9^ First, in each catchment area, we established case-detection systems by mapping and engaging service providers and community informants, covering all sectors of the local health care systems (ie, professional [mental health services], folk [traditional, spiritual healers], and popular [community informants]).^9^ Second, we gave all mental health service professionals, traditional and spiritual healers, and informants materials that described experiences and behaviors characteristic of psychoses, using local terms and language, to facilitate shared understandings.^10^ Third, trained researchers conducted regular checks with mental health professionals, traditional and spiritual healers, and informants to identify potential cases. Finally, in rural villages in Kancheepuram and Ibadan, field workers engaged community informants to identify potential cases. At the end of case ascertainment, we conducted leakage studies by rechecking service registers and completing final checks with mental health professionals, traditional and spiritual healers, and informants. All potential cases were screened using the Screening Schedule for Psychosis. All identified cases were approached to participate in all aspects of INTREPID II. Written informed consent was obtained from those who agreed to participate in the case-control arm of the program; otherwise, ethical approval was obtained from local research ethics committees in each setting to collate basic demographic and clinical information from records (eAppendix 2 in the Supplement).^8^

### Data

Data on sociodemographic characteristics and symptoms, including duration, were collated from cases, relatives, and clinical records (where available) using translated versions of the Medical Research Council Sociodemographic Schedule,^11^ the WHO Personal and Psychiatric History Schedule (PPHS),^12^ and the Schedules for Clinical Assessment in Neuropsychiatry (SCAN).^13^ Duration of untreated psychosis was assessed using the PPHS and defined as the time between onset of psychotic symptoms (ie, symptoms meeting criteria for a rating of 2 [clinically relevant] in the psychosis sections of the SCAN) and date of identification. Date of onset was derived from this, by subtracting the duration of untreated psychosis from age at identification. Diagnoses were determined by consensus based on SCAN data.

Assessments were conducted by researchers fluent in the local language. Researchers underwent extensive training. For relevant assessments, we conducted interrater reliability (IRR) exercises. Researchers rated videos of assessments; these were compared with ratings developed by the principal investigators. For the assessments in this article, researcher ratings were within acceptable margins of principal investigator ratings: SCAN 87% (range, 85%-88%) and PPHS 76% (range, 73%-84%).

### Populations at Risk

We estimated the populations at risk (aged 18-64 years) in each catchment area using the most recent data from official statistics in each country, projected from previous country-wide censuses to the year INTREPID II began or the nearest possible (India: 2011 Census; Nigeria, 2010 Census; Trinidad, 2011 Census). Population data were stratified by age (18-19 years, then 5-year bands), sex, and self-defined ethnic group (ie, Tamil, Hausa, Yoruba, African Trinidadian, Indian Trinidadian, mixed Trinidadian). We multiplied population at risk by period of case ascertainment to estimate person-years at risk (Table 1).^14,15,16,17^

**Table 1.** Information on Population at Risk (Denominator) and Eligible Incidence Cases Identified and Included (Numerator)

Population	Kancheepuram^a^	Ibadan^b^	Trinidad^c^
Denominator			
Population	1 066 319	863 472	720 605
Population age 18-64 y	701 680^d^	624 990	509 905
Start date	01-05-2018	01-05-2018	01-05-2018
End date	30-05-2020	31-07-2020	30-04-2020
Period at risk, mo	25	27	24
Person-years at risk	1 459 495	1 406 227	1 019 809
Numerator			
Screened	1286	911	985
Excluded	1023	774	411
Included	263	137	574
Leakage	5	59	0
Total incidence cases	268	196	574
Cases identified via^e^, %			
Professional sector^f^	44 (16.4)	100 (51.0)	565 (98.4)
Folk sector^f^	0 (0.0)	88 (44.9)	5 (0.9)
Popular sector^f^	224 (83.6)	8 (4.1)	4 (0.7)

^a^ Census of India. Provisional population totals; Tamil Nadu census 2011 subdistrict (Taluk) level.^14^ Totals adjusted to account for projected population growth. Data from 2011 census, with projected increase of 6.9% (6.6% for men and 7.3% for women) to 2020, giving estimated population at risk in 2020. Projections only available at state level (table 17, p 245).^15^

^b^ National Population Commission of Nigeria. Population distribution by sex, state, local government authority, and senatorial district. Abuja, Nigeria: National Population Commission of Nigeria; 2010. Totals adjusted to account for projected rate of population growth. (Data from 2006 census, with projected 3.45% increase year on year, giving estimated population at risk in 2016, for which the latest date projections are available.) Projections only available at state level.^16^

^c^ Central Statistical Office, Ministry of Planning and Sustainable Development. Trinidad and Tobago 2011 Population and Housing Census Demographic Report. Port of Spain, Trinidad: Government of Trinidad and Tobago; 2012. Totals adjusted to accounted for projected rate of population growth. (Data from 2011 census, with projected 2.9% increase to 2020, giving estimated population at risk in 2020.^17^)

^d^ Population aged 18 to 64 years estimated using proportions in this age group for Kancheepuram District (ie, 63% aged 18-64 years; same for men and women).

^e^ χ^2^_4 _= 1200; *P* <.001.

^f^ The professional sector comprises mental health services (public and private), the folk sector comprises traditional and spiritual healers, and the popular sector comprises key informants.

### Statistical Analyses

We compared demographic and clinical characteristics of cases across and within sites using descriptive statistics and univariable tests of association. We used direct standardization to estimate sex- and age-standardized rates of untreated psychoses (per 100 000 person-years) using the World (WHO: 2000-2025) Standard Population (https://seer.cancer.gov/stdpopulations/world.who.html). To test hypotheses relating to differences in rates of untreated psychosis across and within settings, we used Poisson regression to model rate ratios adjusted for sex and age. We fit interaction terms, as appropriate, and tested for interaction using likelihood ratio tests. Statistical analyses were conducted from January 1 to May 1, 2022, using Stata software, version 17 (StataCorp).

## Results

In each catchment area, the population at risk included more than 500 000 individuals (Table 1). Adjusted for duration of case finding, this yielded 3 790 697 total person-years at risk (Kancheepuram: 1 459 495; Ibadan: 1 406 227; Trinidad: 1 019 809). We identified 1038 cases, including 64 through leakage studies (Kancheepuram: 268; median [IQR] age, 42 [33-50] years; 154 women [57.5%]; 114 men [42.5%]; Ibadan: 196; median [IQR] age, 34 [26-41] years; 93 women [47.4%]; 103 men [52.6%]; Trinidad: 574; median [IQR] age, 30 [23-40] years; 235 women [40.9%]; 339 men [59.1%]). Of the 64 cases identified through leakage studies, 5 were identified in Kancheepuram, 59 in Ibadan, and 0 in Trinidad. There were differences between settings in the sources through which cases were identified (χ^2^_4_ = 1200; *P* < .001). In Kancheepuram, most individuals (224 [84%]) were identified via informants (popular sector), with a minority identified via mental health services (professional sector) (44 [16%]) and none via healers (traditional sector). In Ibadan, similar numbers of cases were identified via services (100 [51%]) and healers (88 [45%]), with a small number via informants (8 [4%]). In Trinidad, almost all were identified via services (565 [98%]), with small numbers via healers (5 [1%]) and informants (4 [1%]).

### Demographic and Clinical Characteristics

There were variations across settings in the demographic and clinical profiles of the cohorts (Table 2). For example, there were differences by sex (ie, higher proportion of women in Kancheepuram [58%] compared with Ibadan [47%] and Trinidad [41%]) and by age (ie, higher median age at identification in Kancheepuram [42 years] compared with Ibadan [34 years] and Trinidad [30 years]). These differences are reflected in sex- and age-standardized rates. In Trinidad, the cohort was ethnically heterogeneous (318 African Trinidadian [57%]; 113 Indian Trinidadian [20%]; 130 mixed Trinidadian [23%]). In Kancheepuram (225 Tamil [100%], with data) and Ibadan (2 Hausa [2%] and 125 Yoruba [98%], with data), cohorts were ethnically homogenous. The median (IQR) duration of psychosis was 56 (23-123) months in Kancheepuram, 38 (1-51) months in Ibadan, and 11 (3-27) months in Trinidad. In Kancheepuram, more than 80% of individuals met the criteria for schizophrenia (126 [47%]) or psychosis not otherwise specified (NOS) (112 [42%]). Brief and affective psychoses were rare (10 [approximately 4%]). In Ibadan, approximately one-half of participants (100 [51%]) met criteria for schizophrenia, and approximately one-quarter met criteria for a diagnosis with an affective component (52 [27%] schizoaffective, manic, or depressive psychosis). Few were classified as psychosis NOS (35 [18%]) or brief psychosis (8 [4%]). By contrast, in Trinidad, approximately 40% (221 [39%]) met criteria for schizophrenia, and affective and brief psychosis were more common (176 [31%] schizoaffective, manic, or depressive psychosis; 98 [17%] brief psychosis).

**Table 2.** Demographic and Clinical Characteristics of Untreated Cases by Setting

Characteristic	Kancheepuram (n = 268)	Ibadan (n = 196)	Trinidad (n = 574)	Test statistic	*df*	*P* value
Age at detection, mean (SD)	41.8 (11.5)	35.3 (11.0)	32.7 (11.4)	*F* = 110.0	2	<.001
Median (IQR)	42 (33-50)	34 (26-41)	30 (23-40)	χ^2^ = 53.1	2	<.001
Age at onset,^a^ mean (SD)	35.1 (11.4)	32.1 (11.4)	28.9 (11.8)	*F* = 25.0	2	<.001
Median (IQR)	33 (25-44)	29 (23-38)	26 (20-35)	χ^2^ = 53.1	2	<.001
Onset <18 y, No. (%)^a^						
No	240 (96.4)	185 (94.4)	482 (87.2)	χ^2^ = 21.2	2	<.001
Yes	9 (3.6)	11 (5.6)	71 (12.8)			
Sex						
Men	114 (42.5)	103 (52.6)	339 (59.1)	χ^2^ = 20.2	2	<.001
Women	154 (57.5)	93 (47.4)	235 (40.9)			
Ethnic group,^b^ No. (%)						
Tamil^c^	225 (100.0)	NA	NA	NA	NA	NA
Yoruba^c^	NA	125 (98.4)				
Hausa^c^	NA	2 (1.6)				
Trinidadian						
African	NA	NA	318 (56.7)	NA	NA	NA
Indian			113 (20.1)			
Mixed^d^			130 (23.2)			
DUP,^e^ median (IQR), mo	55.6 (22.8-123.1)	37.8 (1.0-51.3)	11.0 (3.0-26.9)	χ^2^ = 123.6	2	<.001
DUP, dichotomized (1),^e^ No. (%), y						
≤ 2	76 (30.5)	108 (55.1)	406 (73.4)	χ^2^ = 132.3	2	<.001
> 2	173 (69.5)	88 (44.9)	147 (26.6)			
DUP, dichotomized (2),^e^ No. (%), y						
≤ 5	130 (52.2)	161 (82.1)	462 (83.5)	χ^2^ = 96.9	2	<.001
> 5	119 (47.8)	35 (17.9)	91 (16.5)			
*ICD-10* diagnosis, No. (%)						
F20 Schizophrenia	126 (47.0)	100 (51.0)	221 (38.5)	χ^2^ = 282.6	14	<.001
F22 Delusional	15 (5.6)	1 (0.5)	8 (1.4)			
F25 Schizoaffective	4 (1.5)	16 (8.2)	32 (5.5)			
F30-31^f^ Manic	1 (0.4)	26 (13.3)	64 (11.2)			
F32-33^f^ Depressive	8 (3.0)	10 (5.1)	80 (13.9)			
F10-19^f^ Substance use	1 (0.4)	0 (0.0)	21 (3.7)			
F23 Brief	1 (0.4)	8 (4.1)	98 (17.1)			
F28-29^f^ NOS	112 (41.8)	35 (17.9)	50 (8.7)			
Identified via, No. (%)						
Professional^g^	44 (16.4)	100 (51.0)	565 (98.4)	NA	NA	NA
Folk^g^	0 (0.0)	88 (44.9)	5 (0.9)			
Popular^g^	224 (83.6)	8 (4.1)	4 (0.7)			

Abbreviations: DUP, duration of untreated psychosis; *ICD-10*, *International Statistical Classification of Diseases and Related Health Problems*, Tenth Revision (*ICD-10*); NA, not applicable; NOS, not otherwise specified.

^a^ Missing (due to missing DUP): 19 (7%) Kancheepuram; 0 Ibadan; 21 (4%) Trinidad.

^b^ Missing: 43 (16%) Kancheepuram; 69 (35%) Ibadan; 13 (2%) Trinidad.

^c^ Kancheepuram and Ibadan are ethnically homogenous. Kancheepuram is in Tamil Nadu state, in which approximately 90% are fluent Tamil speakers. Ibadan predominantly comprises indigenous Yoruba people. In our sample, in Kancheepuram, 100% (225 of 225 on whom we had data) were Tamil and, in Ibadan, 98% (125 of 127 on whom we had data) were Yoruba.

^d^ Mixed indicates mixed Trinidadian (ie, mixed African/Indian Trinidadian).

^e^ Missing: 19 (7%) Kancheepuram; 0 Ibadan; 21 (4%) Trinidad.

^f^ Psychosis codes only.

^g^ The professional sector comprises mental health services (public and private), the folk sector comprises traditional and spiritual healers, and the popular sector comprises key informants.

### Rates: Site and Diagnosis

**Figure 1.**
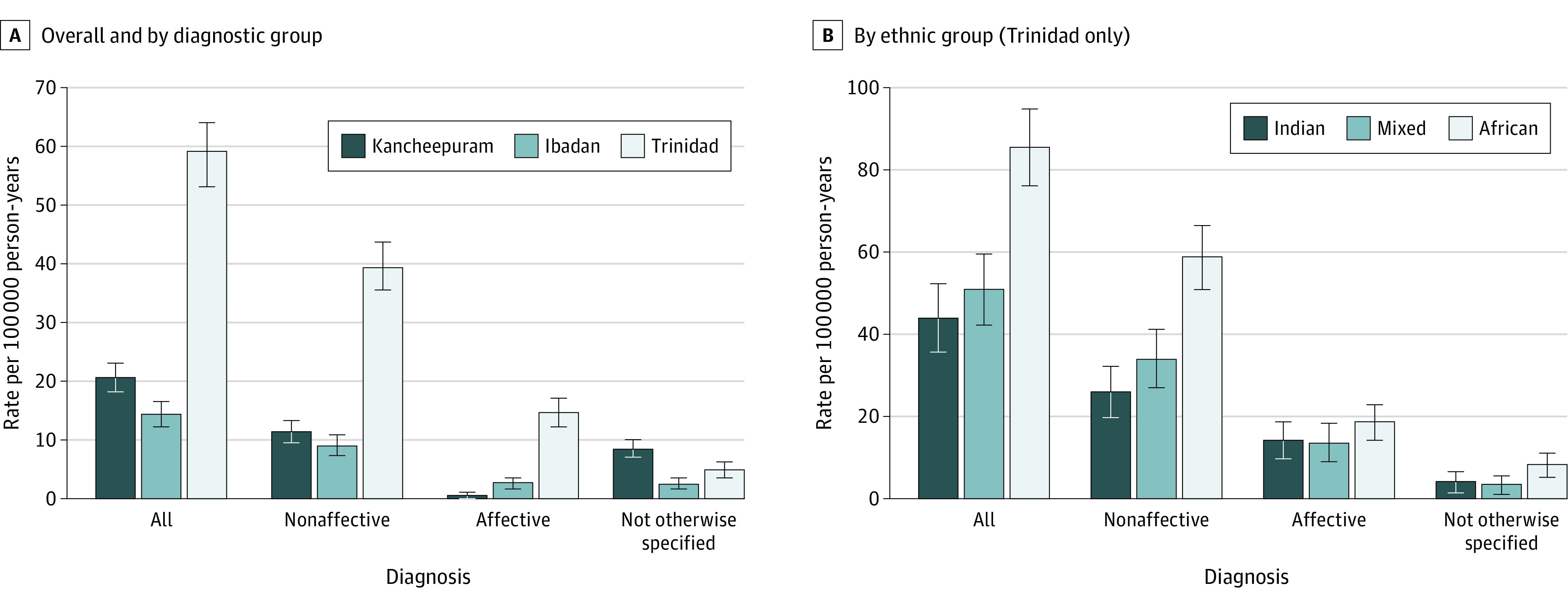
Sex- and Age-Standardized Rates of Untreated Psychoses Overall and by Diagnostic Group and by Ethnic Group (Trinidad Only) Rates of untreated psychoses overall and by diagnostic group (A) and by ethnic group (B). Error bars are 95% CIs. Age is age at detection.

Rates of untreated psychosis varied by setting (Table 3; Figure 1A). The sex- and age-standardized rates of untreated psychoses were 20.7/100 000 person-years (95% CI, 18.2-23.2) in Kancheepuram, 14.4/100 000 person-years (95% CI, 12.3-16.5) in Ibadan, and 59.1/100 000 person-years (95% CI, 54.2-64.0) in Trinidad. Compared with Kancheepuram, the rate in Trinidad was approximately 3 times higher (adjusted IRR [aIRR], 3.03; 95% CI, 2.62-3.51), and the rate in Ibadan was approximately 30% lower (aIRR, 0.71; 95% CI, 0.59-0.85). For nonaffective and affective psychoses, rates were higher in Trinidad, particularly for affective psychoses. In Kancheepuram, there was a relatively high rate of cases categorized as psychosis NOS (8.6/100 000 person-years; 95% CI, 7.0-10.2).

**Table 3.** Sex- and Age (at Detection)-Standardized Rates by Site

Overall	Person-years	No. of cases	(95% CI)	
			Rate^a^	aIRR^b^
All psychoses				
Kancheepuram	1 459 495	268	20.7 (18.2-23.2)	1 [Reference]
Ibadan	1 406 227	196	14.4 (12.3-16.5)	0.71 (0.59-0.85)
Trinidad	1 019 809	574	59.1 (54.2-64.0)	3.03 (2.62-3.51)
Nonaffective				
Kancheepuram	1 459 495	147	11.4 (9.5-13.3)	1 [Reference]
Ibadan	1 406 227	125	9.1 (7.5-10.8)	0.82 (0.64-1.04)
Trinidad	1 019 809	380	39.4 (35.4-43.5)	3.74 (3.09-4.52)
Affective^c^				
Kancheepuram	1 459 495	9	0.7 (0.2-1.1)	0.26 (0.13-0.55)
Ibadan	1 406 227	36	2.7 (1.7-3.6)	1 [Reference]
Trinidad	1 019 809	144	14.7 (12.3-17.2)	5.99 (4.15-8.66)
Psychosis NOS				
Kancheepuram	1 459 495	112	8.6 (7.0-10.2)	1 [Reference]
Ibadan	1 406 227	35	2.6 (1.7-3.5)	0.32 (0.22-0.46)
Trinidad	1 019 809	50	4.9 (3.5-6.4)	0.59 (0.42-0.82)
By ethnic group (Trinidad)				
All psychoses				
Indian	268 813	113	43.9 (35.7-52.2)	1 [Reference]
Mixed^d^	253 081	130	50.7 (42.0-59.5)	1.13 (0.88-1.45)
African	385 437	318	85.4 (76.0-94.9)	1.89 (1.53-2.35)
Nonaffective				
Indian	268 813	67	25.9 (19.6-32.1)	1 [Reference]
Mixed^d^	253 081	88	34.0 (26.9-41.2)	1.28 (0.93-1.76)
African	385 437	219	58.8 (50.9-66.6)	2.18 (1.66-2.86)
Affective^c^				
Indian	268 813	37	14.1 (9.5-18.7)	1 [Reference]
Mixed^d^	253 081	34	13.5 (9.0-18.1)	0.93 (0.58-1.48)
African	385 437	69	18.5 (14.1-22.9)	1.29 (0.86-1.92)
Psychosis NOS				
Indian	268 813	9	4.0 (1.3-6.6)	1 [Reference]
Mixed^d^	253 081	8	3.2 (1.0-5.4)	0.86 (0.33-2.23)
African	385 437	30	8.1 (5.2-11.0)	2.25 (1.07-4.74)

Abbreviations: aIRR, adjusted incidence rate ratio; NOS, not otherwise specified.

^a^ Rate per 100 000 person-years of risk.

^b^ Adjusted for age and sex; modeled using Poisson regression.

^c^ Reference category is Ibadan.

^d^ Mixed indicates mixed Trinidadian (ie, mixed African/Indian Trinidadian).

### Rates: Sex- and Age-Specific

**Figure 2.**
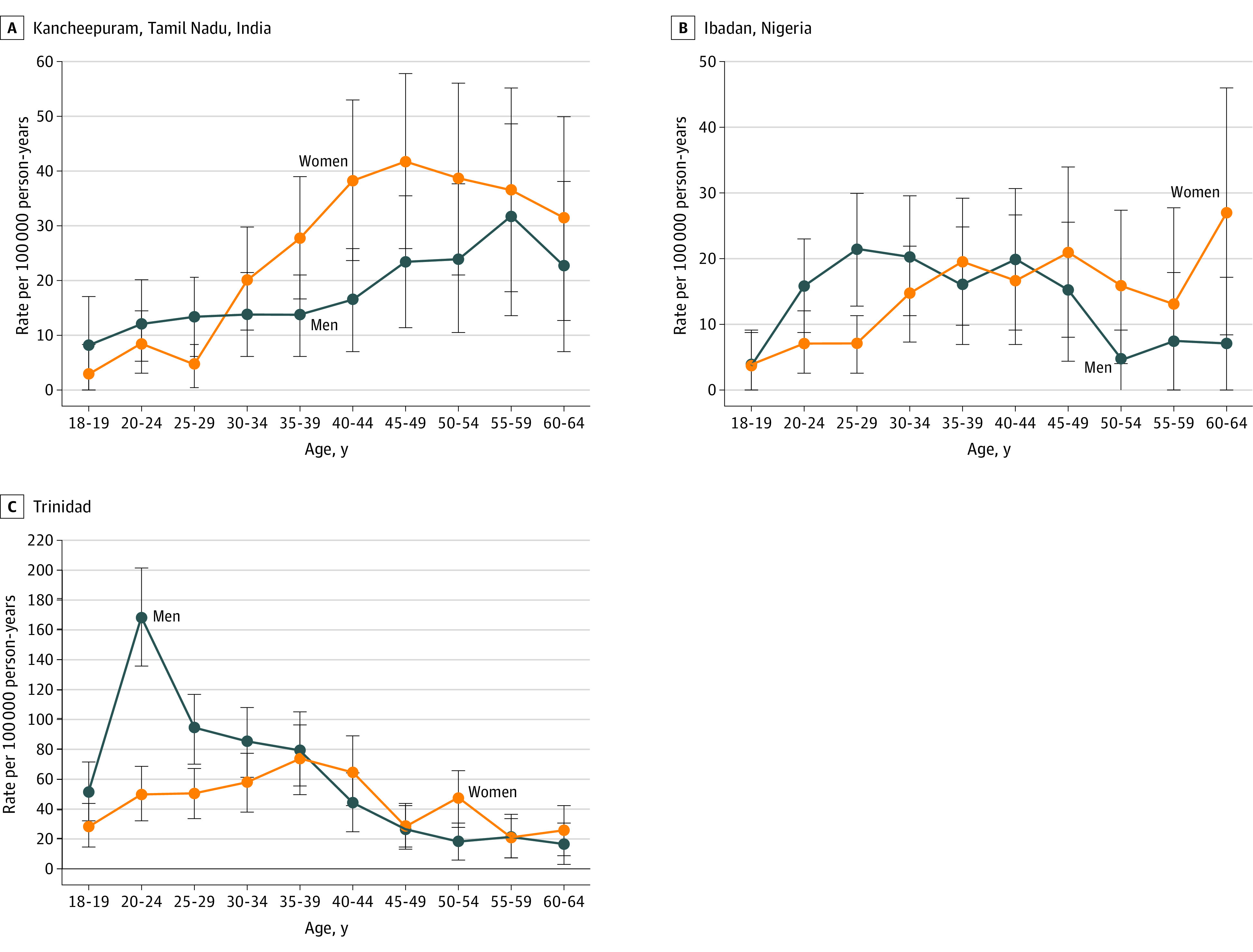
Sex- and Age-Specific Rates of Untreated Psychosis by Site A, Kancheepuram, Tamil Nadu, India (likelihood ratio test for interaction: χ^2^_9_ = 17.12; *P* = .047). B, Ibadan, Nigeria (likelihood ratio test for interaction: χ^2^_9_ = 20.51; *P* = .015). C, Trinidad (likelihood ratio test for interaction: χ^2^_9_ = 44.88; *P* < .001).

Sex- and age-specific rates of untreated psychoses varied by setting (Figure 2; eTables 2-4 in the Supplement). In Kancheepuram, rates were approximately 30% lower among men compared with women (aIRR, 0.73; 95% CI, 0.57-0.93); in Ibadan, rates were more similar among men and women, with at most weak evidence that rates were slightly higher among men (aIRR, 1.21; 95% CI, 0.91-1.60); in Trinidad, rates were approximately 45% higher among men (aIRR, 1.45; 95% CI, 1.23-1.71).

In all settings, differences by sex varied by age at detection, with evidence of sex by age interactions in each setting (Figure 2; eTables 3-4 and 9 in the Supplement). In Kancheepuram, rates were marginally higher among men than women in younger groups (18-29 years) and lower among men in older groups (over 30 years) (likelihood ratio test for interaction: χ^2^_9_ = 17.12; *P* for interaction = .047). In Ibadan, rates were also higher among men than women in younger groups; similar between ages 30 and 49; and lower among men in older groups (likelihood ratio test for interaction: χ^2^_9 _= 20.51; *P* for interaction = .015). In Trinidad, rates were substantially higher among men in the younger groups. From age 35 years, rates were lower for both men and women (likelihood ratio test for interaction: χ^2^_9_ = 44.88; *P* for interaction <.001). These patterns were broadly similar when we used age at onset (eFigure and eTables 5, 6, and 10 in the Supplement).

### Rates: Ethnic Group (Trinidad)

In Trinidad, rates were 2 times higher in the African Trinidadian population (85.4/100 000 person-year; 95% CI, 76.0-94.9) compared with Indian Trinidadian (43.9/100 000 person-year; 95% CI, 35.7-52.2) and mixed populations (50.7/100 000 person-year; 95% CI, 42.0-59.5) (Table 3; Figure 1B). Rates were similarly elevated for African Trinidadian men and women (eTable 7 in the Supplement). When rates for each ethnic group were compared by diagnostic group, the relative increase was greater for nonaffective psychoses (eg, African vs Indian: aIRR, 2.18; 95% CI, 1.66-2.86) than for affective psychoses (eg, African vs Indian: aIRR, 1.29; 95% CI, 0.86-1.92) (Table 3).

### Short Duration Psychosis

Among those with a duration of psychosis less than 2 years, rates were lower in Kancheepuram and, to a lesser extent, in Ibadan compared with Trinidad, reflecting differences across settings in duration of psychosis (ie, sex- and age-standardized rates of [short duration] untreated psychoses: 5.7 [95% CI, 4.4-6.9] in Kancheepuram, 7.8 [95% CI, 6.3-9.4] in Ibadan, 41.6 [95% CI, 37.5-45.7] in Trinidad) (eTable 8 in the Supplement). Consequently, compared with Kancheepuram, the rate of short-duration psychoses in Trinidad was substantially higher (aIRR, 7.68; 95% CI, 6.01-8.92), and the rate in Ibadan was marginally higher (aIRR, 1.32; 95% CI, 0.98-1.77).

## Discussion

INTREPID II is the first program in a generation to investigate the epidemiology, onset, outcomes, and impacts of psychotic disorders in multiple countries in the Global South. Using methods comparable with population-based studies in the Global North, we found considerable heterogeneity in the demographic and clinical profiles and in rates of untreated psychoses in settings in India, Nigeria, and Trinidad.

### Variations in Rates: Place

The patterns of variation in rates of psychoses challenge some accepted assumptions about the epidemiology of psychoses. Age- and sex-standardized rates were approximately 3 times higher in Trinidad than in Kancheepuram and Ibadan. There are 2 sides to this. First, rates in Kancheepuram and Ibadan were relatively low. There are few previous studies for comparison. The 2 that we are aware of in India (Chennai,^18^ Chandigarh^6^) were conducted in the 1980s, and rates of nonaffective psychoses or schizophrenia in these studies were higher (approximately 40-60 per 100 000), albeit the number of cases was small (ie, <125). There are several possible reasons for these differences: methodology, different context, change over time. There are no comparable studies in Nigeria. Second, rates in Trinidad were relatively high. In the European Network of National Schizophrenia Networks Studying Gene-Environment Interactions (EU-GEI) study, the highest rate was similarly approximately 60 per 100 000 in London, UK.^8^ Further, the only other study to publish data on incidence in Trinidad, conducted in the 1990s, reported a lower rate of approximately 20 to 25 per 100 000.^19^ It is possible that rates have increased over time. In this context, it is notable that both substance use and levels of community violence and crime—both of which have been posited as contributory causes of psychoses^20,21,22,23^ have risen markedly in Trinidad since 1999.^24,25^ These increases have been greatest in urban areas, eg, the capital Port of Spain, which forms part of our catchment area.

### Variations in Rates: Diagnosis

It may be that variations in the distribution of diagnoses by setting reflect variations in the distribution of risks in each population. For example, in Trinidad, a context with high levels of trauma and substance use (which have been linked to acute positive and affective symptoms^26,27^), there were relatively high rates of affective and brief psychoses. This said, there is a need for some caution. The low rates of affective and brief psychoses in Kancheepuram and in Ibadan contrast with some previous reports of high rates of brief psychoses in low- and middle-income countries, up to 10 times higher than in high-income countries.^28^

### Variations in Rates: Sex, Age, and Ethnic Group

There were further variations in the sex and age distributions of rates of untreated psychoses by setting. Typically, in studies in the Global North, rates of psychoses tend to be higher among men, particularly in younger age groups.^7,8^ This is what we found in Trinidad. By contrast, the sex and age distributions in Kancheepuram and Ibadan differed from this pattern. In particular, in Kancheepuram, rates were higher among women and in older age groups, a pattern that held when analyses were restricted to those with a short duration of psychoses and when repeated using age at onset. There are possible methodological explanations, eg, bias in case finding, and it is possible that subsequent prospective studies would produce findings more similar to those in high-income countries. This noted, it is also possible that sex and age distributions and profiles vary by context. This makes sense if rates overall are influenced by environmental risks and protective factors. There are, however, very few previous studies for comparison.

In Trinidad, rates were approximately 2 times higher in the African Trinidadian population compared with Indian Trinidadian and mixed. The only previous study in Trinidad did not report rates by ethnic group. Our findings, consequently, need to be considered cautiously and any proposed explanations are speculative. This noted, it may be relevant that there are marked variations by ethnic group in area of residence in Trinidad, with the African population more concentrated in urban settings (eg, Port of Spain: approximately 55% African vs approximately 5% Indian), in which there are higher levels of exposure to established risks for psychosis.

### Limitations

This study has several limitations. First, in each setting we sought to establish case-detection systems tailored to local health contexts. This goes beyond previous studies. However, we still cannot exclude the possibility that we missed cases and that this varied across and within settings. It is possible, for example, that folk and traditional healers and informants more often missed cases. Further, in Kancheepuram, where approximately 10% of the population is non-Tamil, we did not identify any non-Tamil cases. Differential case ascertainment may, therefore, partly explain variations in rates and absence of non-Tamil cases in Kancheepuram.

Second, in line with previous studies, in primary analyses we did not restrict inclusion based on duration of psychosis, and we based age-sex specific rates on age at detection.^6,7,8^ This ensures consistency with previous research and means we can consider the full spectrum of psychotic disorders and of clinical, social, and service-use histories. Still, in the analyses presented here, the variations in duration of psychosis by site mean that there is a need for caution in comparing rates of untreated psychoses, particularly age-sex–specific rates. In sites where duration is relatively long, such as Kancheepuram, the estimated rates may be less accurate proxies for incidence. In this article, we do also provide estimated age-sex–specific rates based on age at onset and estimated rates of untreated psychoses with a duration less than 2 years. However, rates of short-duration psychosis may underestimate incidence, as it may take time for individuals with a psychotic disorder to become visible to detection systems.

Third, in each setting, we relied on projections from previous censuses to estimate populations at risk. We do not know to what extent any inaccuracies in projections varied by site and to what extent, if any, this distorted rate ratios. However, it seems implausible that this could account for large observed differences, eg, between Trinidad and both Kancheepuram and Ibadan. Further, it is in Ibadan, where projections were available only to 2016, that the denominator is most likely underestimated and therefore the rate overestimated.

## Conclusion

Findings of this cohort study add to research that suggests core aspects of psychosis are shaped by historic, economic, and social context. It follows that we can only fully understand the etiology, manifestations, and outcomes of psychoses—indeed the very nature of psychoses—if we research psychoses in context. We cannot assume that what we learn in high-income countries can be generalized elsewhere. In addition, our findings show that the nature and extent of needs for care and support vary across contexts; this points to the necessity of grounding the development and delivery of services in locally contextualized knowledge. Of course, services in all contexts must provide care for a wide range of people. But this does not negate the general point that, on average, needs will vary, and services need to orientate toward this. It is therefore essential that future hypothesis-driven research on psychoses is broadened to encompass a wider range of settings to deepen our understandings of psychoses and of how to deliver humane, accessible, and effective services in diverse contexts.

## Notes

Supplement. **eAppendix 1.** Program Sites **eAppendix 2.** Ethical Approvals **eTable 1. **Economic, Development, and Health Indicators **eTable 2. **Rates of Psychosis, by Sex **eTable 3. **Age (at Detection)–Specific Rates of Psychosis (Men) **eTable 4. **Age (at Detection)–Specific Rates of Psychosis (Women) **eTable 5. **Age (at Onset)–Specific Rates of Psychosis (Men) **eTable 6. **Age (at Onset)–Specific Rates of Psychosis (Women) **eTable 7. **Age- and Sex-Standardized Rates of Untreated Psychoses by Ethnic Group and Sex. (Trinidad Only) **eTable 8. **Age- and Sex-Standardized Rates of All and of Short-Duration Psychosis (for All and Short Duration Cases) **eTable 9. **Rate Ratios for Men vs Women, by Age (at Detection) Category **eTable 10. **Rate Ratios for Men vs Women, by Age (at Onset) Category **eFigure. **Sex- and Age-Specific Rates of Untreated Psychosis by Site
