# Supplementary material for: Epidemiology of Untreated Psychoses in 3 Diverse Settings in the Global South: The International Research Program on Psychotic Disorders in Diverse Settings (INTREPID II)
Source: JAMA Psychiatry. 2022 Nov 16;80(1):40–8. doi: 10.1001/jamapsychiatry.2022.3781 (PMC9669922; doi:10.1001/jamapsychiatry.2022.3781)
Supplement: Supplement. — eAppendix 1. Program Sites eAppendix 2. Ethical Approvals eTable 1. Economic, Development, and Health Indicators eTable 2. Rates of Psychosis, by Sex eTable 3. Age (at Detection)–Specific Rates of Psychosis (Men) eTable 4. Age (at Detection)–Specific Rates of Psychosis (Women) eTable 5. Age (at Onset)–Specific Rates of Psychosis (Men) eTable 6. Age (at Onset)–Specific Rates of Psychosis (Women) eTable 7. Age- and Sex-Standardized Rates of Untreated Psychoses by Ethnic Group and Sex. (Trinidad Only) eTable 8. Age- and Sex-Standardized Rates of All and of Short-Duration Psychosis (for All and Short Duration Cases) eTable 9. Rate Ratios for Men vs Women, by Age (at Detection) Category eTable 10. Rate Ratios for Men vs Women, by Age (at Onset) Category eFigure. Sex- and Age-Specific Rates of Untreated Psychosis by Site [file jamapsychiatry-e223781-s001.pdf]

Supplementary Online Content

Morgan C, Cohen A, Esponda GM, et al; the INTREPID Group. Epidemiology of untreated psychoses in 3 diverse settings in the Global South: the international research program on psychotic disorders in diverse settings (INTREPID II). *JAMA Psychiatry*. Published online November 16, 2022. doi:10.1001/jamapsychiatry.2022.3781

eAppendix 1. Program Sites

eAppendix 2. Ethical Approvals

eTable 1. Economic, Development, and Health Indicators

eTable 2. Rates of Psychosis, by Sex

eTable 3. Age (at Detection)–Specific Rates of Psychosis (Men)

eTable 4. Age (at Detection)–Specific Rates of Psychosis (Women)

eTable 5. Age (at Onset)–Specific Rates of Psychosis (Men)

eTable 6. Age (at Onset)–Specific Rates of Psychosis (Women)

eTable 7. Age- and Sex-Standardized Rates of Untreated Psychoses by Ethnic Group and Sex. (Trinidad Only)

eTable 8. Age- and Sex-Standardized Rates of All and of Short-Duration Psychosis (for All and Short Duration Cases)

eTable 9. Rate Ratios for Men vs Women, by Age (at Detection) Category

eTable 10. Rate Ratios for Men vs Women, by Age (at Onset) Category

eFigure. Sex- and Age-Specific Rates of Untreated Psychosis by Site

This supplementary material has been provided by the authors to give readers additional information about their work.

© Morgan C et al. *JAMA Psychiatry.*

eAppendix 1. Program Sites

The programme sites were selected to maximise diversity and potential for comparisons both between and within sites and with existing data sets. The sites span three continents, are economically, socially, and culturally diverse, and are in countries undergoing rapid economic and social change (Supplementary Table 1). In each site, we identified catchment areas that comprise urban and rural areas with populations of around 500,000 adults aged 18-64 years. In India, the catchment area comprises 4 taluks, or sub-districts, of the Kancheepuram District (Chengelpattu, Thiruporur, Uthiramerur, and Maduranthakam), which is directly south of Chennai in Tamil Nadu, southern India. The area is mainly rural, with several urban centres, and has a total population of around 1,000,000 (based on projections from the 2011 Census to 2020), with population densities ranging from ~ 361–737 per km^2. In Nigeria, the catchment area comprises 3 local government areas in and around the city of Ibadan in Oyo State (Ibadan North East, Ibadan South East and Ona-Ara), which is north of Lagos in the south-west of Nigeria. The area comprises urban (Ibadan North East, Ibadan South East) and semi-rural (Ona Ara) areas and has a total population of around 900,000 (based on projections from the 2010 Census to 2016, the latest year for which projections are available), with population densities ranging from ~ 914–18,356 per km^2, with some areas of very high density reflecting the fact that Ibadan is the third most populous city in Nigeria. In Trinidad, the catchment area comprises 7 municipalities (Port of Spain, Arima, Chaguanas, Tunapuna-Piarco, San Juan-Laventille, Diego Martin, and Sangre Grande) in the northern half of the island. The area comprises urban and rural areas and has a total population of around 720,000 (based on projections from the 2011 Census to 2020), with population densities ranging from ~ 82–3,090 per km^2. Kancheepuram and Ibadan are ethnically homogenous. Kancheepuram is in Tamil Nadu state, in which around 90% are native Tamil speakers, with a range of other groups comprising around 10% (e.g., Telugu, Kannada). Ibadan predominantly comprises indigenous Yoruba people (~ 96%). Trinidad is ethnically heterogenous, in which around 38% are African-Trinidadian, 36% Indian Trinidadian, and 25% mixed, with a small number of other groups (e.g., White, Chinese) comprising around 1%.

eAppendix 2. Ethical Approvals

Ethical approvals for all aspects of the programme were provided by ethics review boards at each lead institution and, where necessary, by other relevant bodies. In the UK: King's College London, UK (Reference: HR-17/18-5601); London School of Hygiene and Tropical Medicine, UK (Reference: 15807). In India: Health & Family Welfare Department, Government of Tamil Nadu (Letter No.14248/EAPI-2/2018-2, Dated 28.05.2018); Institutional Ethics Committee of SCARF (Date of issue: 28.11.2017); Institutional Ethics Committee, Madras Medical College (No.19082019, Dated 06.08.2019). In Nigeria: University of Ibadan and University College Hospital Ethics Committee (Registration number: NHREC/05/01/2008a; Study number: UI/EC/18/0099), Ibadan, Nigeria; Institute for Advanced Medical Research & Training (IAMRAT), College of Medicine, Ibadan, Nigeria). In Trinidad, Eastern (Reference: PHO: 24/1), North Central (Reference: 185-43 CD), and North West (approved on 9 July 2018, no reference number issued) Regional Health Authorities.

© Morgan C et al. *JAMA Psychiatry.*

eTable 1. Economic, Development, and Health Indicators

	India		Nigeria		Trinidad and Tobago
	National	Tamil Nadu†	National	Oyo State††	National†††
Urban population (a, b) (2020)	34.9% (2.0% increase since 2010)	48.4% (2011)	52.0% (8.5% increase since 2010)	no data^	53.2% (8.8% increase since 2010)
Projected annual rate of urbanization (a) (2015 to 2020, estimated)	2.4 %	no data**	3.9%	no data^	0.2%
Economic (GDP) growth (d)					
2010 to 2019 (average per year)	6.7%	7.2%	3.7%	no data	- 0.3%
2020	- 8.0%	2.0%	- 1.8%	no data	- 7.8%
Poverty (c) (i.e. living on less than $1.90 per day) (2020)	22.5%	11.3%	39.1%	no data	0%
Income Gini‡ Coefficient (c) (2010)	37.8	no data	43.0	no data	no data
Human Development Index (c) (2020)					
Index	0.65	no data	0.54	no data	0.80
Rank	131	-	161	-	61
Infant mortality per 1,000 live births (c) (2020)	29.9	15.0	75.7	no data	16.4
Life expectancy at birth (years) (c) (2020)	69.7	71.7	54.7	no data	73.5
Suicide Rate per 100,000 (c) (2020)					
Men	18.5	no data	17.5	no data	
Women	14.5	no data	17.1	no data	
Homicide Rate per 100,000 (c) (2020)	3.1	no data	34.5	no data	30.6
Literacy, 15+ years (c) (2020)					
Overall	74.4%	80.1% (Urban 87.0%) (Rural 73.5%)	62.0%	no data	98.7%

† Our catchment area in India is in Tamil Nadu state

†† Our catchment area in Nigeria is in Oyo state

††† There are no data available for regions in Trinidad and Tobago

© Morgan C et al. *JAMA Psychiatry.*

** Information on projected rate of urbanisation not available. Between the 1991 and 2011 census, the urban population in Tamil Nadu grew by 14.3% (from 34.2% to 48.4%)

^ Directly comparable information on urban populations in Oyo State not available. Ibadan is the third largest city in Nigeria (after Lagos and Kano), with (at 2006 census) a population of around 2,338,659

(a) World Urbanisation Prospectus, 2011 Revision. United Nations (http://esa.un.org/unpd/wup/CD-ROM/Urban-Rural-Population.htm)

(b) 2011 Indian Census

(c) Human Development Report 2020 (http://hdr.undp.org/en)

(d) The World Bank (http://data.worldbank.org/indicator/NY.GDP.MKTP.KD.ZG)

© Morgan C et al. *JAMA Psychiatry.*

eTable 2. Rates of Psychosis, by Sex

	Person years at risk	Cases	Rate *	95% CI	RR **	95% CI
All psychoses						
Kancheepuram						
women	681,291	154	24.1	20.3-28.0	1.00	-
men	683,368	114	17.2	14.1-20.4	0.73	0.57-0.93
Ibadan						
women	733,220	93	14.2	11.2-17.3	1.00	-
men	673,008	103	14.6	11.7-17.5	1.21	0.91-1.60
Trinidad						
women	512,170	235	48.2	42.0-54.5	1.00	-
men	507,640	339	70.0	62.5-77.6	1.45	1.23-1.71
Non-affective						
Kancheepuram						
women	681,291	90	14.1	11.2-17.0	1.00	-
men	683,368	57	8.7	6.4-11.0	0.62	0.45-0.87
Ibadan						
women	733,220	59	8.8	6.4-11.2	1.00	-
men	673,008	66	9.4	7.1-11.8	1.22	0.86-1.73
Trinidad						
women	512,170	115	23.9	19.5-28.3	1.00	-
men	507,640	265	55.0	48.3-61.7	2.32	1.86-2.88
Affective						
Kancheepuram						
women	681,291	5	0.7	0.1-1.4	1.00	-
men	683,368	4	0.6	0.11.1	0.78	0.21-2.92
Ibadan						
women	733,220	21	3.3	1.8-4.8	1.00	-
men	673,008	15	2.0	1.0-3.1	0.79	0.40-1.53
Trinidad						
women	512,170	97	19.8	15.8-23.8	1.00	-

© Morgan C et al. *JAMA Psychiatry.*

men	507,640	47	9.7	6.9-12.5	0.49	0.34-0.69
Psychosis NOS						
Kancheepuram						
women	681,291	59	9.3	6.9-11.7	1.00	-
men	683,368	53	8.0	5.8-10.1	0.89	0.61-1.28
Ibadan						
women	733,220	13	2.1	0.9-3.3	1.00	-
men	673,008	22	3.2	1.8-4.5	1.87	0.94-3.71
Trinidad						
women	512,170	23	4.6	2.6-6.5	1.00	-
men	507,640	27	5.3	3.3-7.4	1.18	0.68-2.06

* Rate per 100,000 person years of risk

** Adjusted for age and sex; modelled using Poisson regression

RR Rate Ratio

© Morgan C et al. *JAMA Psychiatry.*

eTable 3. Age (at Detection)–Specific Rates of Psychosis (Men)

	Kancheepuram				Ibadan				Trinidad			
Age	**Person years at risk**	**Cases**	**Rate ***	**95% CI**	**Person years at risk**	**Cases**	**Rate ***	**95% CI**	**Person years at risk**	**Cases**	**Rate ***	**95% CI**
18-19	37,032	3	8.1	0.1-17.3	54,359	2	3.7	0.1-8.8	51,861	27	52.1	32.4-71.7
20-24	98,857	12	12.1	5.3-19.0	120,426	19	15.8	8.7-22.9	60,278	101	167.6	134.9-200.2
25-29	103,461	14	13.5	6.4-20.6	112,479	24	21.3	12.8-29.9	67,069	63	93.9	70.7-117.1
30-34	93,564	13	13.9	6.3-21.4	93,357	19	20.4	11.2-29.5	58,827	50	85.0	61.4-108.5
35-39	86,206	12	13.9	6.0-21.8	75,587	12	15.9	6.9-24.9	50,067	40	79.9	55.1-104.6
40-44	72,984	12	16.4	7.1-25.7	65,616	13	19.8	9.0-30.6	44,815	20	44.6	25.1-64.2
45-49	64,170	15	23.4	11.5-35.2	53,130	8	15.1	4.6-25.5	50,907	14	27.5	13.1-41.9
50-54	50,277	12	23.9	10.4-37.4	43,228	2	4.6	0.1-11.0	47,813	9	18.8	6.5-31.3
55-59	41,331	13	31.5	14.4-48.5	26,958	2	7.4	0.1-17.7	40,878	9	22.0	7.6-36.4
60-64	35,486	8	22.5	6.9-38.2	27,868	2	7.2	0.1-17.1	35,125	6	17.1	3.4-30.7

* Rate per 100,000 person years of risk

© 2022 Morgan C et al. *JAMA Psychiatry.*

eTable 4. Age (at Detection)–Specific Rates of Psychosis (Women)

	Kancheepuram				Ibadan				Trinidad			
Age	**Person years at risk**	**Cases**	**Rate ***	**95% CI**	**Person years at risk**	**Cases**	**Rate ***	**95% CI**	**Person years at risk**	**Cases**	**Rate ***	**95% CI**
18-19	34,782	1	2.9	0.1-8.5	53,313	2	3.8	0.1-9.0	51,546	15	29.1	14.4-43.8
20-24	104,170	9	8.6	3.0-14.3	136,313	10	7.3	2.8-11.9	61,144	31	50.7	32.9-68.5
25-29	110,928	5	4.5	0.6-8.5	142,135	10	7.0	2.7-11.4	68,116	34	49.9	33.1-66.7
30-34	88,700	18	20.3	10.9-29.7	108,610	16	14.7	7.5-21.9	57,285	33	57.6	38.0-77.3
35-39	86,664	24	27.7	16.6-38.8	82,151	16	19.5	9.9-29.0	49,343	36	73.0	49.1-96.8
40-44	68,005	26	38.2	23.5-52.9	65,676	11	16.7	6.9-26.6	45,968	30	65.3	41.9-88.6
45-49	62,551	26	41.6	25.6-57.5	47,903	10	20.9	7.9-33.8	51,355	15	29.2	14.4-44.0
50-54	49,260	19	38.6	21.2-55.9	44,545	7	15.7	4.1-27.4	48,931	23	47.0	27.8-66.2
55-59	41,160	15	36.4	18.0-54.9	22,991	3	13.0	0.1-27.8	43,203	9	20.8	7.2-34.4
60-64	35,071	11	31.4	12.8-49.9	29,583	8	27.0	8.3-45.8	35,279	9	25.5	8.8-42.2

* Rate per 100,000 person years of risk

© 2022 Morgan C et al. *JAMA Psychiatry.*

eTable 5. Age (at Onset)–Specific Rates of Psychosis (Men)

	Kancheepuram				Ibadan				Trinidad			
Age	**Person years at risk**	**Cases**	**Rate ***	**95% CI**	**Person years at risk**	**Cases**	**Rate ***	**95% CI**	**Person years at risk**	**Cases**	**Rate ***	**95% CI**
18-19	37,032	4	10.8	0.2-21.4	54,359	6	11.01	2.2-19.9	51,861	48	92.6	66.4-118.7
20-24	98,857	26	26.3	16.2-36.4	120,426	24	19.9	12.0-27.9	60,278	89	147.6	117.0-178.3
25-29	103,461	16	15.5	7.9-23.0	112,479	24	21.3	12.8-29.9	67,069	50	74.6	53.9-95.2
30-34	93,564	19	20.3	11.2-29.4	93,357	19	20.4	11.2-29.5	58,827	44	74.8	52.7-96.9
35-39	86,206	9	10.4	3.6-17.3	75,587	10	13.2	5.0-21.4	50,067	22	43.9	25.6-62.3
40-44	72,984	6	8.2	1.6-14.8	65,616	4	6.1	0.1-12.1	44,815	12	26.8	11.6-41.9
45-49	64,170	6	9.4	1.9-16.8	53,130	4	7.5	0.2-14.9	50,907	8	15.7	4.8-26.6
50-54	50,277	9	17.9	6.2-29.6	43,228	2	4.6	0.1-11.0	47,813	6	12.5	2.5-22.6
55-59	41,331	5	12.1	1.5-22.7	26,958	2	7.4	0.1-17.7	40,878	4	9.8	0.2-19.4
60-64	35,486	2	5.6	0.1-13.4	27,868	1	3.6	0.1-10.6	35,125	2	5.7	0.1-13.6

* Rate per 100,000 person years of risk

© 2022 Morgan C et al. *JAMA Psychiatry.*

eTable 6. Age (at Onset)–Specific Rates of Psychosis (Women)

	Kancheepuram				Ibadan				Trinidad			
Age	**Person years at risk**	**Cases**	**Rate ***	**95% CI**	**Person years at risk**	**Cases**	**Rate ***	**95% CI**	**Person years at risk**	**Cases**	**Rate ***	**95% CI**
18-19	34,782	4	11.5	0.2-22.8	53,313	4	7.5	0.2-14.9	51,546	14	27.2	12.9-41.4
20-24	104,170	18	17.3	9.3-25.3	136,313	14	10.3	4.9-15.7	61,144	26	42.5	26.2-58.9
25-29	110,928	18	16.2	8.7-23.7	142,135	19	13.4	7.4-19.4	68,116	36	52.9	35.6-70.1
30-34	88,700	25	28.2	17.1-39.2	108,610	14	12.9	6.1-19.6	57,285	28	48.9	30.8-67.0
35-39	86,664	25	28.8	17.5-40.2	82,151	11	13.4	5.5-21.3	48,343	35	70.9	47.4-94.4
40-44	68,005	15	22.1	10.9-33.2	65,676	8	12.2	3.7-20.6	45,968	18	39.2	21.1-57.2
45-49	62,551	17	27.2	14.3-40.1	47,903	4	8.4	0.2-16.5	51,355	12	23.4	10.1-36.6
50-54	49,260	8	16.2	5.0-27.5	44,545	5	11.2	1.4-21.1	48,931	14	28.6	13.6-43.6
55-59	41,160	7	17.0	4.4-29.6	22,991	7	30.4	7.9-53.0	43,203	10	23.1	8.8-37.5
60-64	35,071	1	2.9	0.1-8.4	29,583	2	6.8	0.1-16.1	35,279	4	11.3	0.2-22.4

* Rate per 100,000 person years of risk

© 2022 Morgan C et al. *JAMA Psychiatry.*

eTable 7. Age- and Sex-Standardized Rates of Untreated Psychoses by Ethnic Group and Sex. (Trinidad Only)

	Person Years	Cases	Rate *	95% CI	RR **	95% CI
All psychoses						
Men						
Indian	134,798	67	53.3	40.4-66.2	1.00	-
Mixed	121,083	81	63.8	49.9-77.8	1.18	0.85-1.63
African	193,375	185	99.6	85.2-114.0	1.83	1.38-2.42
Women						
Indian	134,015	46	34.6	24.4-44.8	1.00	-
Mixed	131,998	49	37.6	27.0-48.1	1.05	0.70-1.57
African	192,062	133	71.2	59.0-83.4	2.00	1.43-2.80

* Rate per 100,000 person years of risk

** Adjusted for age and sex; modelled using Poisson regression

RR Rate Ratio

© 2022 Morgan C et al. *JAMA Psychiatry.*

eTable 8. Age- and Sex-Standardized Rates of All and of Short-Duration Psychosis (for All and Short Duration Cases)

	All cases	Rate (1) *	95% CI	RR **	95% CI	Short DUP cases ^	Rate (2) *	95% CI	RR **	95% CI
All psychoses										
Kancheepuram	268	20.7	18.2-23.2	1.00	-	76	5.7	4.4-6.9	1.00	-
Ibadan	196	14.4	12.3-16.5	0.71	0.59-0.85	108	7.8	6.3-9.4	1.32	0.98-1.77
Trinidad	574	59.1	54.2-64.0	3.03	2.62-3.51	406	41.6	37.5-45.7	7.68	6.01-8.92

(1) All cases

(2) Recent onset (i.e., duration of psychosis of less than 2 years) cases only

* Rate per 100,000 person years of risk

^ Duration of psychosis less than 2 years

** Adjusted for age and sex; modelled using Poisson regression

RR Rate Ratio

© 2022 Morgan C et al. *JAMA Psychiatry.*

eTable 9. Rate Ratios for Men vs Women, by Age (at Detection) Category

	Kancheepuram		Ibadan		Trinidad	
Age	**Rate Ratio (men vs. women)**	**95% CI**	**Rate Ratio (men vs. women)**	**95% CI**	**Rate Ratio (men vs. women)**	**95% CI**
18-19	2.82	0.29-27.09	0.98	0.14-6.96	1.79	0.95-3.36
20-24	1.40	0.59-3.33	2.15	1.00-4.63	3.30	2.21-4.94
25-29	3.00	1.08-8.33	3.03	1.45-6.34	1.88	1.24-2.86
30-34	0.68	0.34-1.40	1.38	0.71-2.69	1.48	0.95-2.29
35-39	0.50	0.25-1.01	0.82	0.39-1.72	1.10	0.70-1.72
40-44	0.43	0.22-0.85	1.18	0.53-2.64	0.68	0.39-1.20
45-49	0.56	0.30-1.06	0.72	0.28-1.83	0.94	0.45-1.95
50-54	0.62	0.30-1.27	0.29	0.06-1.42	0.40	0.19-0.87
55-59	0.86	0.41-1.81	0.57	0.10-3.40	1.06	0.42-2.66
60-64	0.72	0.29-1.79	0.27	0.06-1.25	0.67	0.24-1.88

Likelihood Ratio Tests: χ^2 and p-values for interactions by age category and sex:

- Kancheepuram: χ^2 17.12, df 9, p 0.047

- Ibadan: χ^2 20.51, df 9, p 0.015

- Trinidad: χ^2 44.88, df 9, p < 0.001

© 2022 Morgan C et al. *JAMA Psychiatry.*

eTable 10. Rate Ratios for Men vs Women, by Age (at Onset) Category

	Kancheepuram		Ibadan		Trinidad	
Age	**Rate Ratio (men vs. women)**	**95% CI**	**Rate Ratio (men vs. women)**	**95% CI**	**Rate Ratio (men vs. women)**	**95% CI**
18-19	0.94	0.23-3.76	1.47	0.42-5.21	3.40	1.88-6.18
20-24	1.53	0.83-2.78	1.94	1.00-3.75	3.47	2.24-5.37
25-29	0.95	0.49-1.87	1.60	0.87-2.91	1.41	0.92-2.16
30-34	0.72	0.40-1.31	1.47	0.75-2.90	1.53	0.95-2.46
35-39	0.36	0.17-0.78	1.09	0.45-2.61	0.62	0.36-1.06
40-44	0.37	0.14-0.96	0.50	0.15-1.66	0.68	0.33-1.42
45-49	0.34	0.14-0.87	1.13	0.30-4.20	0.67	0.27-1.65
50-54	1.10	0.43-2.86	0.41	0.08-2.12	0.44	0.17-1.14
55-59	0.71	0.23-2.24	0.24	0.05-1.17	0.42	0.13-1.35
60-64	1.98	0.18-21.80	0.53	0.05-5.85	0.50	0.09-2.74

Likelihood Ratio Tests: χ^2 and p-values for interactions by age category and sex:

- Kancheepuram: χ^2 16.49, df 9, p 0.057

- Ibadan: χ^2 12.87, df 9, p 0.169

- Trinidad: χ^2 57.18, df 9, p < 0.001

© 2022 Morgan C et al. *JAMA Psychiatry.*

eFigure. Sex- and Age-Specific Rates of Untreated Psychosis by Site

(a) Kancheepuram, Tamil Nadu, India

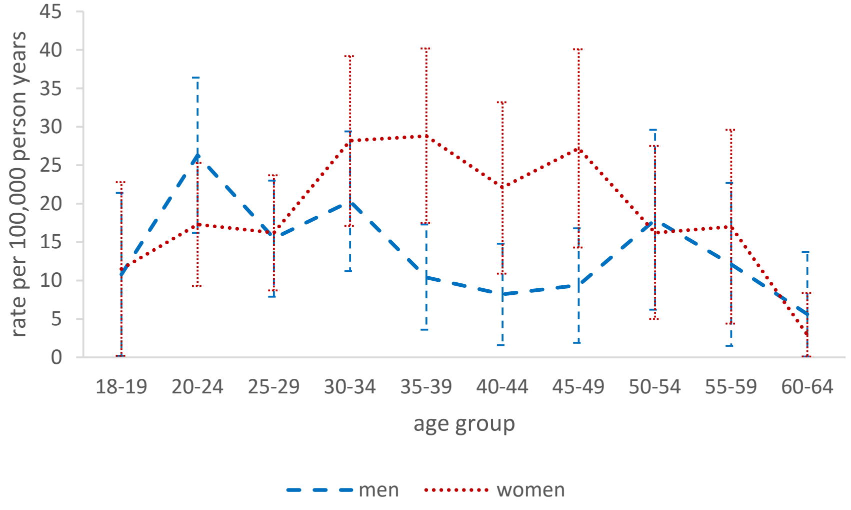

Likelihood Ratio Test for interaction: χ^2 16.49, df 9, p 0.057

(b) Ibadan, Nigeria

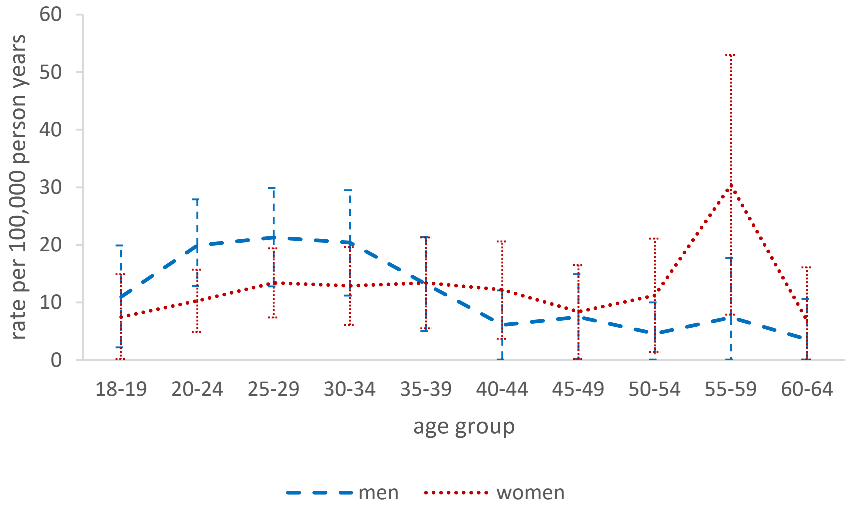

Likelihood Ratio Test for interaction: χ^2 12.87, df 9, p 0.169

(c) Trinidad

© 2022 Morgan C et al. *JAMA Psychiatry.*

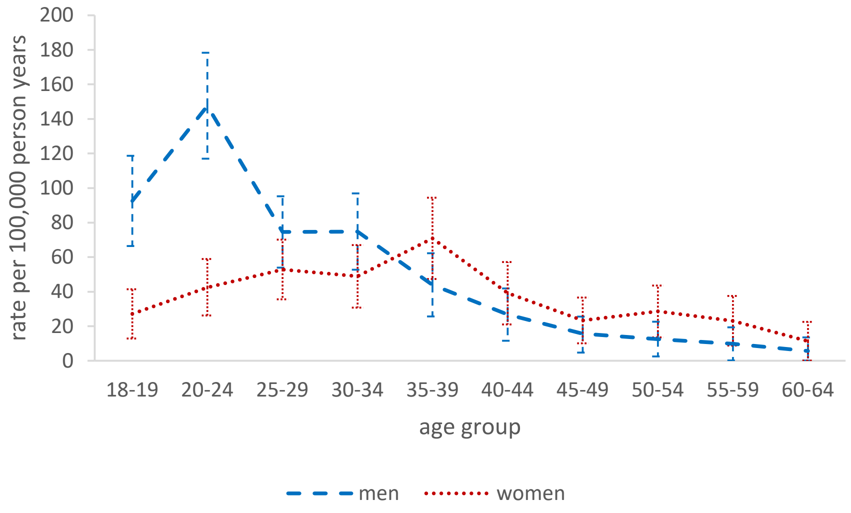

Likelihood Ratio Test for interaction: χ^2 57.18, df 9, $p < 0.001$

© 2022 Morgan C et al. *JAMA Psychiatry.*
